# Efficacy evaluation of Buyang Huanwu Decoction in the treatment of ischemic stroke in the recovery period: A systematic review of randomized controlled trials

**DOI:** 10.3389/fphar.2022.975816

**Published:** 2022-10-14

**Authors:** Raoqiong Wang, Junhao Ren, Shuangyang Li, Xue Bai, Wubin Guo, Sijin Yang, Qibiao Wu, Wei Zhang

**Affiliations:** ^1^ Faculty of Chinese Medicine and State Key Laboratory of Quality Research in Chinese Medicine, Macau University of Science and Technology, Macau, China; ^2^ National Traditional Chinese Medicine Clinical Research Base of the Affiliated Traditional Chinese Medicine Hospital of Southwest Medical University, Luzhou, China; ^3^ The Affiliated Traditional Chinese Medicine Hospital of Southwest Medical University, Luzhou, China; ^4^ Zhuhai MUST Science and Technology Research Institute, Zhuhai, China

**Keywords:** Buyang Huanwu Decoction, ischemic stroke, recovery period, systematic review, meta-analysis, traditional Chinese medicine

## Abstract

**Background and purpose:** Buyang Huanwu decoction (BYHWD) is widely used in the treatment of ischemic stroke in the recovery period**,** and many clinical trials have been reported, but its clinical efficacy and safety have not been fully evaluated. In this study, we conducted a systematic review and meta-analysis to evaluate the clinical efficacy and safety of BYHWD in the recovery period.

**Materials and methods:** Eight databases, including CNKI, Wanfang Database, VIP Database, China Biomedical Literature Database, PubMed, Cochrane Library, EMBASE, and Web of Science, were searched from the establishment of the database to 13 April 2022. We selected all eligible randomized controlled trials of BYHWD in the treatment of ischemic stroke during the recovery period. Systematic review and meta-analysis were conducted in accordance with PRISMA (Preferred Reporting Items for Systematic Reviews and Meta-Analysis) guidelines. The National Institutes of Health Stroke Score (NIHSS) was the primary outcome, and the Chinese Stroke Scale (CSS), activities of daily living (ADL), and adverse drug reaction (ADR) were the secondary outcomes.

**Results:** A total of 39 randomized controlled trials were included, and 3,683 patients in the recovery period of ischemic stroke were recruited. Compared with conventional treatment alone, BYHWD combined with conventional treatment significantly decreased the NIHSS score (MD = -1.44, 95% CI: 1.75, -1.12, *p* < 0.00001), the CSS score (MD = -1.18, 95% CI: 2.02, -0.34, *p* = 0.006), improved the ADL (MD = 4.33, 95% CI: 3.06, 5.61, *p* < 0.00001), and did not increase the adverse reactions of patients (OR = 0.88, 95% CI: 0.48, 1.61, *p* = 0.67).

**Conclusion:** BYHWD is an effective and safe therapy for the recovery of ischemic stroke. To further determine the efficacy and safety of BYHWD in the treatment of ischemic stroke in the recovery period, more high-quality, multicenter, and prospective RCTs are needed.

## 1 Introduction

Ischemic stroke is a cerebrovascular disease caused by cerebral ischemia and hypoxia due to cerebral blood supply disorder, resulting in necrosis, softening, and the formation of infarction (Feske, 2021). Ischemic stroke is divided into the acute phase, recovery phase, and sequelae phase. The recovery period refers to 2 weeks to 6 months after the onset of the disease. This period is the key period for the recovery of patients, and it is also an important part of clinical treatment (Belova and Bogdanov, 2021). Patients with ischemic stroke are prone to neurological dysfunction, slow recovery, and many complications, which seriously affect their physical and mental health (Stinear et al., 2020). Active and effective treatments for stroke patients during the recovery period can significantly improve their daily living ability. Besides rehabilitation, antiplatelet aggregation and anticoagulant therapy are commonly used western medicine treatments for these patients, but they easily cause drug resistance and adverse reactions (Boulanger et al., 2018). In recent years, traditional Chinese medicine (TCM) has shown a good effect on ischemic stroke in the recovery period (Guo et al., 2020; Liu, et al., 2021).

Ischemic stroke belongs to the category of “stroke” in TCM. It is mostly caused by deficiency of Qi and blood, lack of nourishment for the brain, block of phlegm and blood stasis, obstruction of brain and collaterals, deficiency of liver and kidney or hyperactivity of liver yang, and disturbance of wind and yang, which in turn causes hemiplegia, skewed tongue, hemianopia, aphasia and other symptoms (Zhao and Zhao, 2021). The main pathogenesis of ischemic stroke is characterized by “wind, fire, phlegm, Qi and blood stasis”. Wang Qingren pioneered the theory of “Qi deficiency and blood stasis” and believed that “the loss of vitality is its source” and “if vitality is deficient, it will not reach the blood vessels. Once the blood vessels have no gas, the blood will stop and become stasis” (Wang, 1999). It should be treated by supplementing qi, activating blood circulation, and removing blood stasis (Zhai et al., 2022). BYHWD is a classical prescription for regulating Qi and blood, removing phlegm and blood stasis in TCM (Shao et al., 2022). It is composed of *Astragalus* trimestris L [Fabaceae, the dried root of *Astragalus* trimestris L]; Angelica sinensis (Oliv.) Diels [Apiaceae, the dried root of Angelica sinensis (Oliv.) Diels]; Paeonia officinalis subsp. Officinalis [Paeoniaceae, the dried root and rhizome of Paeonia officinalis subsp. Officinalis]; Pheretima aspergillum (E. Perrier) [Megascolecidae, the dried body of Pheretima aspergillum (E. Perrier)]; Oreocome striata (DC.) Pimenov and Kljuykov [Apiaceae, the dried rhizome of Pheretima aspergillum (E. Perrier) ]; Oreocome striata (DC.) Pimenov and Kljuykov]; Curcuma longa L [Zingiberaceae, the dried flower of Curcuma longa L]; Prunus persica (L.) Batsch [the dried seed of Prunus persica (L.) Batsch], according to the ratio 120: 6: 5: 3: 3: 3: 3. In a previous study, an HPLC-DAD-ELSD method was developed for simultaneous determination of 12 bioactive compounds in BYHWD, including calycosin-O-β-D-glucoside ononin, calycosin, astragaloside IV and astragaloside I from Radix Astragalis; tetramethylpyrazine, ferulic acid and Z-ligustilide from Radix Angelicae Sinensis and Rhizoma Ligustici Chuanxiong; hydroxysafflor yellow A and kaempferol from Flos Carthami; paeoniflorin from Radix paeoniae Rubra; and amygdalin from Semen persicae. (Liu et al., 2010). In another study, Wang *et al.* found that hydroxysafflor yellow A, astragaloside IV, ferulic acid, ligustrazine, Z-ligustilide, and linoleic acid were considered to be bioactive compounds of BYHWD (Wang et al., 2021). Kaempferol, Quercetin, Mairin, Jaranol, Hederagenin and AstragalosideIV are the compounds of *Astragalus* trimestris L; Baicalein, Quercetagetin,Beta-carotene, and Baicalin are the compounds of Paeonia officinalis subsp. Officinalis and Curcuma longa L; Ferulic acid and Cis-ligustilide are the compounds of Angelica sinensis (Oliv.) Diels; Ligustrazine and Z-ligustilide are the compounds of Oreocome striata (DC.) Pimenov and Kljuykov; Sitosterol alpha1 and Folinic acid are the compounds of Prunus persica (L.) Batsch; Arachidonic acid and Dihydrocapsaicin are the compounds of Pheretima aspergillum (E. Perrier) (Table 1).

**TABLE 1:** Components of buyang huanwu decoction.

Scientific name	Family	English name	Chinese name	Part used	Quantity (gram)
Astragalus trimestris L	Fabaceae	Astragalus membranaceus	Huang Qi	Root	120
Angelica sinensis (Oliv.) Diels	Apiaceae	Chinses angelica	Dang Gui	Root	6
Paeonia officinalis subsp. Officinalis	Paeoniaceae	Paeonia rubra	Chi Shao	Root and rhizome	5
Pheretima aspergillum (E. Perrier)	Megascolecidae	Earthworm	Di Long	Dried Body	3
Oreocome striata (DC.) Pimenov and Kljuykov	Apiaceae	Sargentgloryvine	Chuan Xiong	Rhizome	3
Curcuma longa L	Zingiberaceae	safflower	Hong Hua	Flower	3
Prunus persica (L.) Batsch	Rosaceae	Peach kernel	Tao Ren	Seed	3

Many previous studies have shown that BYHWD has a good therapeutic effect on ischemic stroke. Based on network pharmacology, Wang K et al. found that the active ingredients of Buyang Huanwu Decoction in the treatment of ischemic stroke are baicalein *β*- Carotene, baicalin, kaempferol, *etc.* (Wang K, 2021). Cai GX *et al.* studied the effects of BYHWD on neurological function, quality of life, and serum vascular endothelial growth factor (VEGF) in convalescent patients with cerebral infarction in a randomized controlled trial (RCT), showing that BYHWD can improve the neurological function and quality of life of convalescent patients with cerebral infarction and increase serum VEGF (Cai and Liu, 2010). Jin C et al. conducted a meta-analysis to evaluate the role of BYHWD in poststroke fatigue patients. The results showed that BYHWD could improve the fatigue severity scale score and the total clinical effective rate (Jin et al., 2021). In recent years, BYHWD has been widely used in the treatment of ischemic stroke in the recovery period, and a large number of clinical trials have described its efficacy and safety, but no rigorous clinical research can provide reliable clinical evidence. The sample size of these trials is generally not large, and it is difficult to convince the public that BYHWD has a significant effect in the treatment of ischemic stroke in the recovery period based on the results of small sample data, which limits the use and promotion of BYHWD to a certain extent. In addition, no systematic review or meta-analysis has focused on the clinical efficacy and safety of BYHWD in the recovery period of ischemic stroke. Therefore, in this study, we conducted a systematic review and meta-analysis to evaluate the clinical efficacy and safety of BYHWD in the recovery period of ischemic stroke.

## 2 Methods

We conducted this systematic review and meta-analysis in accordance with the PRISMA (preferred Reporting Item for Systematic Reviews and Meta-Analyses) guidelines (Liberati et al., 2009).

### 2.1 Search strategy

Two independent reviewers (Wang and Ren) searched CNKI, Wanfang Database, VIP Database, China Biomedical Literature Database, PubMed, Cochrane Library, EMBASE, and Web of Science. The last search date was 13 April 2022. The search terms used were (“Apoplexy” OR “Stroke” OR “Cerebral Infarction” OR “Brain Infarction” OR “Ischemic Stroke” OR “Ischemic Apoplexy” OR “Cerebrovascular accident” AND “Buyang Huanwu Decoction” AND “random” OR “randomized controlled trial” OR “controlled clinical trial” OR (RCT) OR (RCT) OR (RCTs). No restrictions were imposed on language or publication status.

### 2.2 Type of study

#### 2.2.1 Inclusion criteria

1) Type of study: A randomized controlled trial using integrated traditional Chinese and Western medicine in the recovery period of ischemic stroke. The languages are limited to Chinese and English. 2) Research subjects: patients were diagnosed with ischemic stroke in the recovery period (2 weeks to 6 months after onset). 3) Interventions: The control group received conventional treatment (including controlling blood pressure, improving microcirculation, expanding cerebral vessels, using neurotrophic agents and physical therapy, *etc.* The experimental group was given BYHWD on the basis of conventional treatment. 4) Outcomes: Studies including the National Institute of Health Stroke Scale (NIHSS), Chinese Stroke Scale (CSS), Activities of daily living (ADL), and Adverse drug reaction (ADR).

### 2.2.2 Exclusion criteria

1) Nonrandomized controlled trials or studies that do not indicate the type of study. 2) The included literature can only extract part of the original data, which makes the data impossible to extract. 3) Animal experiments, literature reviews, conference papers. 4) Outcomes studies that did not include NIHSS, CSS, ADL, and ADR. 5) Studies where interventions did not meet the requirements.

### 2.3 Participant characteristics

Age, sex, and race were not the limiting conditions for the inclusion criteria. As long as the ischemic stroke patients in the recovery period who met the above criteria were considered to meet the inclusion criteria.

### 2.4 Interventions

This study selected all RCTs comparing BYHWD with conventional methods in the treatment of ischemic stroke in the recovery period, regardless of language, publication status, or blinding method. Nonrandomized trials, reviews, case reports, and animal studies were excluded. The experimental group used both BYHWD and CT, and the control group used CT alone for comparison. Conventional treatment was the same in both groups.

### 2.5 Outcome measures

The NIHSS was the primary outcome measure, and CSS, ADL, and ADR were the secondary outcomes.

NIHSS (National Institute of Health Stroke Scale) score, which is a quantitative indicator of the severity of AIS disease, is often used as a surrogate endpoint in clinical research and stratifies patients according to the NIHSS score to guide clinical decision-making (Yamal, 2021). It is divided into 11 items, including consciousness, gaze, visual field, facial paralysis, upper limb movement, lower limb movement, ataxia, sensation, language, dysarthria, and neglect, with a score of 0–42. The lower the score, the better the neurological function. The Chinese Stroke Scale (CSS) is based on the standard evaluation of clinical efficacy revised by the fourth national Cerebrovascular Disease Conference (The Fourth National Academic Conference on cerebrovascular disease, 1996). Efficacy standards: A sensory test is performed on the big toe. Symptom score: yes = 1, no = 0; reflex score: none = 2, diminished = 1L, normal = 0; sensory test score: abnormal = 1, normal = 0. Among them, six points are from symptoms, eight points are from the reflexes of both lower extremities, and five points are from the sensation of the thumbs. The total score is added up, from normal = 0 points to the highest score of 19 points. The patient’s ability to do daily living (ADL) was assessed by the Basel index, with a total score of 0–100. A score <40 points indicated that the patient had severe activity disorder; 41–60 points, indicated that the patient needed help to complete daily activities. 60 points meant that the patient needed some help to complete daily activities. The higher the score, the stronger the ADL (Strini et al., 2020). Adverse drug reaction (ADR) mainly referred to gastrointestinal reactions after taking drugs, such as nausea and retching. The internal consistency reliability of NIHSS, CSS, and ADL was high, and the three scales had common validity, but the predictive validity of CSS and ADL was not as comprehensive as NIHSS (Wang et al., 1999; Wu, 2007; Tao 2009).

### 2.6 Data extraction

Two researchers (Wang and ren) independently screened the literature in strict accordance with the inclusion and exclusion criteria. First, the literature was initially screened by reading the title and abstract and then further screened by reading the full text. In case of disagreement, a third party (Wu and Zhang) judged, and finally decided to include or exclude through discussion. Then, two researchers (Li and Bai) independently extracted and included relevant research data, including title, author, year, country, diagnosis method of ischemic stroke, the sample size of each group, age, sex, treatment method, treatment time, outcome indicators and evaluation methods, and main research results.

### 2.7 Methodological quality assessment

The authenticity of the RCTs was assessed by two investigators (Guo and Bai) according to the Cochrane Handbook, and the risk of bias in the literature was assessed according to the Cochrane Risk of Bias Tool. In case of disagreement, a third party (Zhang and Yang) was consulted. The risk of bias was assessed using seven criteria, including random sequence generation, concealed assignment, blinding of participants and personnel, incomplete outcome data, selective reporting, and other biases. The risk of bias was classified into three categories: “low” (+), “high” (-), and “unclear” (?). The Jadad scale quality score was used to evaluate the methodological quality of the literature, with one to two points for low quality and three to five points for high quality.

### 2.8 Data synthesis and analysis

Revman 5.4 software was used for meta-analysis (Copenhagen: the Nordic Cochrane Centre, the Cochrane Collaboration, 2014). Pooled effect size: Count data and measurement data were analyzed by odds ratio (OR), relative risk (RR), mean difference (MD), or standard mean difference (SMD). Heterogeneity analysis: I^2^ was used to assess the heterogeneity of the studies. When I^2^ < 50%, *p* > 0.1, it indicated that the heterogeneity was not significant, and a fixed effect model was used; when I^2^ ≥ 50%, *p* ≤ 0.1, it indicated that the heterogeneity was substantial, and the source of heterogeneity needed to be analyzed. If there was substantial heterogeneity, a random effect model was used (Cumpston and Li, 2019; Wang et al., 2019; Chen H. et al., 2020; Wu et al., 2020); if the heterogeneity still existed, the source of heterogeneity was analyzed from both methodological and clinical aspects, and subgroup analysis was used.

### 2.9 Risk of bias across trials

Funnel plots and Egger’s test were carried out to examine the potential bias in the included trials when the number of RCTs was ≥10 (Egger et al., 1997; Wu et al., 2020).

### 2.10 Quality of evidence

Two independent reviewers (Wang and Ren) used the GRADE (grading of recommendations, assessment, development, and evaluations) method (Guyatt et al., 2008) to evaluate the risk of bias in each included trial. If there was disagreement on the downgrade or upgrade evaluation, it was evaluated and decided by the third party (Wu and Zhang). Evidence evaluation adopts four grades of “high”, “medium”, “low” and “extremely low”.

## 3 Results

### 3.1 Study identification and selection

**FIGURE 1:**
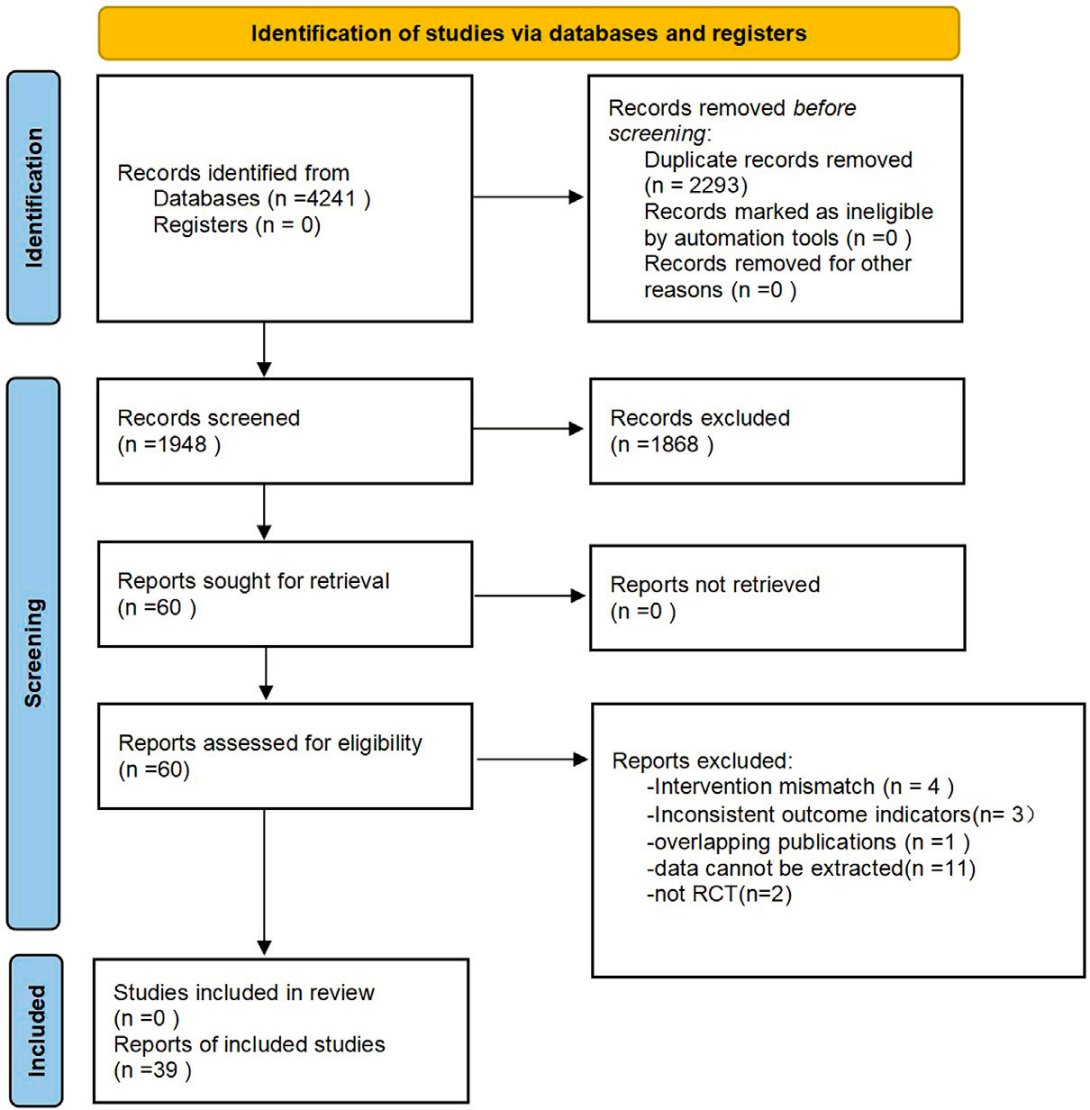
PRISMA diagram of searching.

After searching major databases, a total of 4,241 articles were retrieved. A total of 2294 duplicates were excluded, and 1948 remained; 1868 were excluded after reading the title and abstract, and 60 remained; 21 were excluded after reading the full text, and 39 were finally included in the study. Figure 1 shows the general flow of the study selection process. Table 2 summarizes the general characteristics of the 39 studies.

**TABLE 2:** Characteristics of studies included in the meta-analysis.

Serial number	References	Race	Design	Sample size (T/C)	Age (years) (T/C)	Male/female ratio (T; C)	Outcome measure(s)	Interventions		Treatment duration
								Treatment group	Control group	
1	Zuo and Lin (2020)	East Asia	RCT	50 (25/25)	67.01 ± 2.13/67.21 ± 2.21	14/11; 15/10	NIHSS, CSS, ADL	BYHWD plus CT	CT	2 weeks
2	Zhen Jia Quan, 2016	East Asia	RCT	98 (49/49)	56.4 ± 7.6/56.8 ± 7.2	28/21; 29/20	CSS, ADL	BYHWD plus CT	CT	4 weeks
3	Zhang (2017)	East Asia	RCT	100 (50/50)	59.87 ± 4.64/60.46 ± 4.92	30/20; 31/19	NIHSS, ADR	BYHWD plus CT	CT	3 months
4	Zhang (2013)	East Asia	RCT	100 (50/50)	62.7 (45–76)	53/43	NIHSS	BYHWD plus CT	CT	2months
5	Zhang (2018)	East Asia	RCT	180 (90/90)	71.4 ± 4.7	Not reported	TCM, ADL	BYHWD plus CT	CT	4 weeks
6	Zhang and Xiong (2020)	East Asia	RCT	78 (39/39)	65.32 ± 5.74/65.67 ± 5.21	21/18; 20/19	NIHSS	BYHWD plus CT	CT	3 months
7	Yu (2013)	East Asia	RCT	102 (49/53)	Not reported	Not reported	ADL	BYHWD plus CT	CT	6months
8	Yang (2018)	East Asia	RCT	60 (30/30)	56.14 ± 3.18/55.31 ± 2.27	17/13; 16/14	NIHSS	BYHWD plus CT	CT	4 weeks
9	Yang (2020)	East Asia	RCT	80 (40/40)	63.25 ± 4.68/63.99 ± 4.71	24/16; 23/17	NIHSS	BYHWD plus CT	CT	4 weeks
10	Yang et al. (2021)	East Asia	RCT	90 (45/45)	58.69 ± 7.52/58.72 ± 7.58	30/15; 28/17	NIHSS	BYHWD plus CT	CT	2months
11	Xue and Xue (2015)	East Asia	RCT	74 (36/38)	Not reported	Not reported	CSS	BYHWD plus CT	CT	15d
12	Xu et al. (2017)	East Asia	RCT	170 (85/85)	65.33 ± 6.72/65.16 ± 7.05	45/40; 47/38	TCM, ADL, NIHSS	BYHWD plus CT	CT	6 weeks
13	Xu (2014)	East Asia	RCT	110 (60/50)	62.1 ± 11.6/62.3 ± 10.6	37/23; 31/19	CSS, ADL	BYHWD plus CT	CT	3 weeks
14	Xiang (2019)	East Asia	RCT	60 (30/30)	64.18 ± 3.44/64.24 ± 3.53	18/12; 17/13	NIHSS, ADL	BYHWD plus CT	CT	Not reported
15	Wu (2021)	East Asia	RCT	66 (33/33)	63.35 ± 4.20/63.42 ± 4.25	18/15; 20/13	NIHSS, ADL, ADR	BYHWD plus CT	CT	1month
16	Wang et al. (2021)	East Asia	RCT	92 (46/46)	56.70 ± 5.33/56.39 ± 5.50	26/20; 27/19	NIHSS, ADL	BYHWD plus CT	CT	4 weeks
17	Wang et al. (2012)	East Asia	RCT	96 (54/42)	61.2 ± 11.7/62.5 ± 9.6	35/19; 22/20	CSS, ADL	BYHWD plus CT	CT	4 weeks
18	Sui (2014)	East Asia	RCT	120 (60/60)	63.89 ± 7.05/64.06 ± 8.73	33/27; 34/26	NIHSS	BYHWD plus CT	CT	60d
19	Shi (2016)	East Asia	RCT	60 (30/30)	61.84 ± 3.13/61.25 ± 3.22	17/13; 18/12	ADR	BYHWD plus CT	CT	4 weeks
20	Shang (2021)	East Asia	RCT	72 (36/36)	59.63 ± 7.32/58.76 ± 7.45	23/13; 20/16	NIHSS, ADL	BYHWD plus CT	CT	14d
21	Meng et al. (2014)	East Asia	RCT	120 (60/60)	64.58 ± 7.63/65.37 ± 7.80	36/24; 33/27	ADL	BYHWD plus CT	CT	3 weeks
22	Liu (2018)	East Asia	RCT	30 (15/15)	62.7 ± 4.5/63.1 ± 4.2	8/7; 9/6	NIHSS	BYHWD plus CT	CT	21d
23	Lin (2014)	East Asia	RCT	84 (41/43)	Not reported	Not reported	ADL, ADR	BYHWD plus CT	CT	12 weeks
24	Li (2012)	East Asia	RCT	130 (65/65)	61.3 ± 10.8/61.1 ± 11.2	35/15; 36/14	CSS	BYHWD plus CT	CT	4 weeks
25	Li (2018)	East Asia	RCT	70 (35/35)	61.8 ± 5.3/62.3 ± 5.1	19/16; 18/17	NIHSS	BYHWD plus CT	CT	30d
26	Li and Wei (2021)	East Asia	RCT	78 (39/39)	69.32 ± 5.66/68.95 ± 5.53	29/10; 28/11	NIHSS, ADL	BYHWD plus CT	CT	2 weeks
27	Li (2014)	East Asia	RCT	84 (41/43)	Not reported	Not reported	ADL, ADR	BYHWD plus CT	CT	12 weeks
28	Li (2011)	East Asia	RCT	61 (31/30)	45–70/47–72	21/10; 19/11	NIHSS	BYHWD plus CT	CT	14d
29	Li (2019)	East Asia	RCT	84(42/42)	54.34 ± 8.22/54.52 ± 8.16	23/19; 24/18	NIHSS, ADL, ADR	BYHWD plus CT	CT	4 weeks
30	Li and Li (2010)	East Asia	RCT	112(56/56)	59.1 ± 5.1/57.9 ± 6.5	31/25; 30/26	NIHSS, ADL, ADR	BYHWD plus CT	CT	30d
31	Jin and Xu (2019)	East Asia	RCT	60(30/30)	60.3 ± 7.2/58.6 ± 7.5	18/12; 16/14	NIHSS	BYHWD plus CT	CT	2months
32	Jiang (2019)	East Asia	RCT	100(50/50)	57.9 ± 1.1/57.8 ± 1.2	28/22; 29/21	NIHSS	BYHWD plus CT	CT	4 weeks
33	Ji (2016)	East Asia	RCT	60(30/30)	73.1 ± 11.9/72.5 ± 1.25	17/13; 18/12	ADL,ADR	BYHWD plus CT	CT	4 weeks
34	Han (2014)	East Asia	RCT	80(40/40)	64.58 ± 7.63/65.37 ± 7.80	36/24; 33/27	CSS, ADL	BYHWD plus CT	CT	4 weeks
35	Fang et al. (2019)	East Asia	RCT	76 (38/38)	66.5 ± 4.5/65.9 ± 5.2	20/18; 24/14	NIHSS	BYHWD plus CT	CT	4 weeks
36	Du (2018)	East Asia	RCT	90(45/45)	65.18 ± 2.24/65.12 ± 2.13	23/22; 24/21	CSS, ADR	BYHWD plus CT	CT	4 weeks
37	Diao (2017)	East Asia	RCT	60(30/30)	64.15 ± 5.65/63.45 ± 5.15	20/10; 20/10	NIHSS	BYHWD plus CT	CT	2months
38	Chen Yan, 2016	East Asia	RCT	70(35/35)	42–75	39/31	ADL	BYHWD plus CT	CT	8 weeks
39	Chen Xiao Bing, 2018	East Asia	RCT	376(188/188)	60.2 ± 8.3/111 ± 77	112/76	NI HSS, ADL	BYHWD plus CT	CT	Not reported

Notes: 1. RCT: randomized controlled trial; 2. T/C: Treatment group/control group; 3. NIHSS: national institute of health stroke scale; CSS: cincinnati stroke scale; ADL: activities of daily living; ADR: adverse drug reaction; 4. INTERVENTION STUDY; treatment group; BYHWD, buyang huanwu decoction or modified buyang huanwu decoction; 5. Control group: CT, conventional treatment (including controlling blood pressure, improving microcirculation, expanding cerebral vessels, using neurotrophic agents and physical therapy).

### 3.2 Risk of bias

**FIGURE 2:**
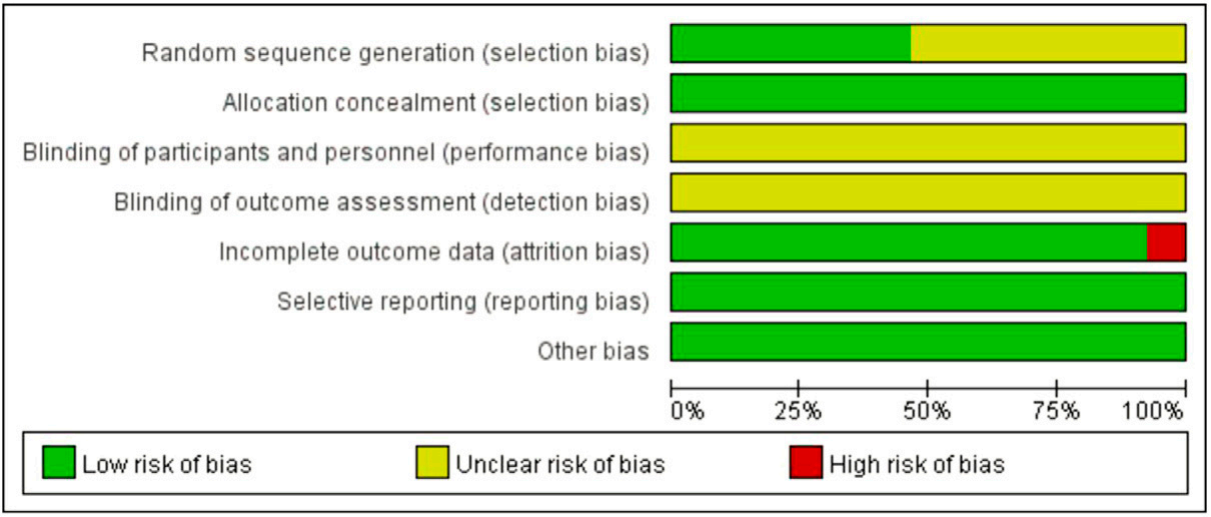
Risk of bias graph.

**FIGURE 3:**
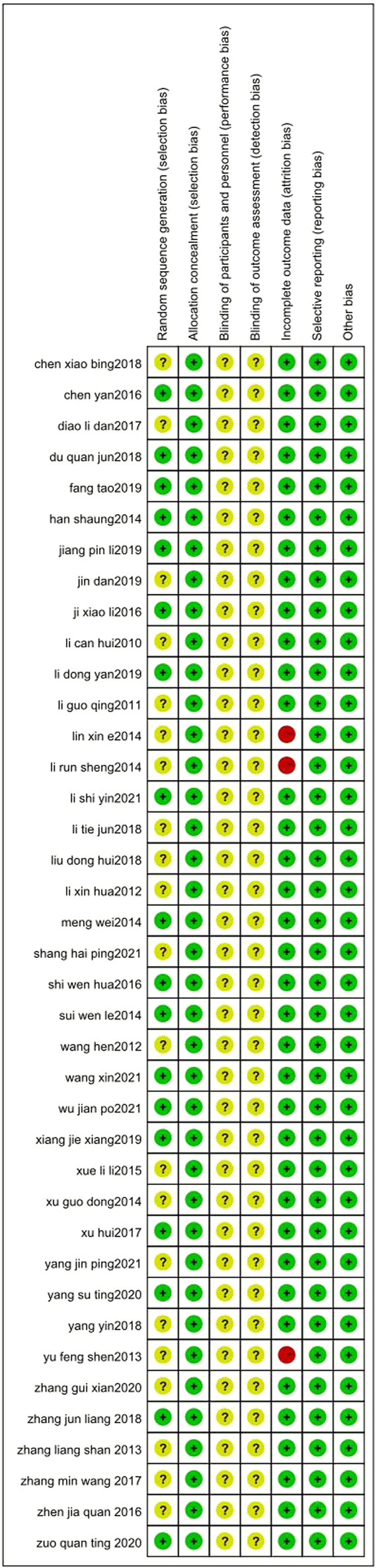
Risk of bias summary.

The risks of bias in the trials are shown in Table 3 and Figure 2, and Figure 3. All 39 trials included in this study explicitly used random sequence generation, of which 18 described the randomization methods in detail (random number table method for 16 trials: Liang et al., 2021; Yang 2022; Zuo and Lin, 2020; Zhang, 2018; Xu et al.,2017; Xiang, 2019; Wu, 2021; Wang et al., 2021; Sui, 2014; Shi, 2016; Li and Wei, 2021; Li, 2019; Jiang, 2019; Ji, 2016; Han, 2014; Fang et al., 2019; Chen and Cao, 2016; random alphabet method for one trial: (Meng et al., 2014); random envelope method for one trial: (Du, 2018). The other 21 articles described the use of randomization but did not provide detailed information on the methods of randomization.

**TABLE 3:** The methodological quality of the included trials assessed using the Cochrane Risk of Bias Tool.

Serial number	References	Random sequence generation	Allocation concealment	Blinding of participants and personnel	Blinding of outcome assessment	Incomplete outcome data	Selective reporting	Other bias
1	Zuo and Lin (2020)	+	+	?	?	+	+	+
2	Zhen Jia Quan, 2016	?	+	?	?	+	+	+
3	Zhang (2017)	?	+	?	?	+	+	+
4	Zhang (2013)	?	+	?	?	+	+	+
5	Zhang (2018)	+	+	?	?	+	+	+
6	Zhang and Xiong (2020)	?	+	?	?	+	+	+
7	Yu (2013)	?	+	?	?	-	+	+
8	Yang (2018)	?	+	?	?	+	+	+
9	Yang (2020)	+	+	?	?	+	+	+
10	Yang et al. (2021)	?	+	?	?	+	+	+
11	Xue and Xue (2015)	?	+	?	?	+	+	+
12	Xu et al. (2017)	+	+	?	?	+	+	+
13	Xu (2014)	?	+	?	?	+	+	+
14	Xiang (2019)	+	+	?	?	+	+	+
15	Wu (2021)	+	+	?	?	+	+	+
16	Wang et al. (2021)	+	+	?	?	+	+	+
17	Wang et al. (2012)	?	+	?	?	+	+	+
18	Sui (2014)	+	+	?	?	+	+	+
19	Shi (2016)	+	+	?	?	+	+	+
20	Shang (2021)	?	+	?	?	+	+	+
21	Meng et al. (2014)	+	+	?	?	+	+	+
22	Liu (2018)	?	+	?	?	+	+	+
23	Lin (2014)	?	+	?	?	-	+	+
24	Li (2012)	?	+	?	?	+	+	+
25	Li (2018)	?	+	?	?	+	+	+
26	Li and Wei (2021)	+	+	?	?	+	+	+
27	Li (2014)	?	+	?	?	-	+	+
28	Li (2011)	?	+	?	?	+	+	+
29	Li (2019)	+	+	?	?	+	+	+
30	Li and Li (2010)	?	+	?	?	+	+	+
31	Jin and Xu (2019)	?	+	?	?	+	+	+
32	Jiang (2019)	+	+	?	?	+	+	+
33	Ji (2016)	+	+	?	?	+	+	+
34	Han (2014)	+	+	?	?	+	+	+
35	Fang et al. (2019)	+	+	?	?	+	+	+
36	Du (2018)	+	+	?	?	+	+	+
37	Diao (2017)	?	+	?	?	+	+	+
38	Chen and Cao (2016)	+	+	?	?	+	+	+
39	Chen and Zhao (2018)	?	+	?	?	+	+	+

+ = low risk of bias; ? = unclear risk of bias; - = high risk of bias.

Based on the information from the included literature, all the studies performed allocation concealment. The blinding of participants or personnel and the blinding of outcome assessments were not mentioned in any of the studies. Detection bias on complete outcome data was considered low in all trials. All data were also considered to be at low risk of selective reporting and other biases.

In addition, the Jadad scale quality score is shown in Table 4. The final scores of 39 articles were all three or above, belonging to high-quality literature.

**TABLE 4:** The methodological quality of the included trials assessed using the Jadad scale quality score.

Serial number	References	Random sequence generation	Double-blind method	Withdrawals and missed visits	Score
1	Zuo and Lin (2020)	2	1	1	4
2	Zhen Jia Quan, 2016	1	1	1	3
3	Zhang (2017)	1	1	1	3
4	Zhang (2013)	1	1	1	3
5	Zhang (2018)	2	1	1	4
6	Zhang and Xiong (2020)	1	1	1	3
7	Yu (2013)	1	1	1	3
8	Yang (2018)	1	1	1	3
9	Yang (2020)	2	1	1	4
10	Yang et al. (2021)	1	1	1	3
11	Xue and Xue (2015)	1	1	1	3
12	Xu et al. (2017)	2	1	1	4
13	Xu (2014)	1	1	1	3
14	Xiang (2019)	2	1	1	4
15	Wu (2021)	2	1	1	4
16	Wang et al. (2021)	2	1	1	4
17	Wang et al. (2012)	1	1	1	3
18	Sui (2014)	2	1	1	4
19	Shi (2016)	2	1	1	4
20	Shang (2021)	1	1	1	3
21	Meng et al. (2014)	2	1	1	4
22	Liu (2018)	1	1	1	3
23	Lin (2014)	1	1	1	3
24	Li (2012)	1	1	1	3
25	Li (2018)	1	1	1	3
26	Li and Wei (2021)	2	1	1	4
27	Li (2014)	1	1	1	3
28	Li (2011)	1	1	1	3
29	Li (2019)	2	1	1	4
30	Li and Li (2010)	1	1	1	3
31	Jin and Xu (2019)	1	1	1	3
32	Jiang (2019)	2	1	1	4
33	Ji (2016)	2	1	1	4
34	Han (2014)	2	1	1	4
35	Fang et al. (2019)	2	1	1	4
36	Du (2018)	2	1	1	4
37	Diao (2017)	1	1	1	3
38	Chen and Cao (2016)	2	1	1	4
39	Chen and Zhao (2018)	1	1	1	3

1–2 points for low quality; three to five points for high quality.

### 3.3 Outcome measures

The summary of the meta-analysis is presented in Table 5.

**TABLE 5:** Summary of the meta-analysis.

Outcome or subgroup	No. Of studies	No. Of participants	Statistical method	Effect size	p
Adverse drug reaction	12	956	RR (fixed), 95% CI	0.88 [0.50, 1.57]	0.67
			OR (fixed), 95% CI	0.88 [0.48, 1.61]	0.67
			RD (fixed), 95% CI	-0.01 [-0.03, 0.02]	0.68
Activities of daily living	20	2194	SMD (fixed), 95% CI	0.27 [0.19, 0.36]	<0.00001^a^
			WMD (fixed), 95% CI	4.33 [3.06, 5.61]	<0.00001^a^
National Institute of Health Stroke Scale	23	2121	SMD (fixed), 95% CI	-0.38 [-0.47, -0.29]	<0.00001^a^
			WMD (fixed), 95% CI	-1.44 [-1.75, -1.12]	<0.00001^a^
Cincinnati Stroke Scale	6	568	SMD (fixed), 95% CI	-0.23 [-0.39, -0.06]	0.007^a^
			WMD (fixed), 95% CI	-1.18 [-2.02, -0.34]	0.006^a^

a The treatment group had significantly improved outcomes.

OR, odds ratio; RD, risk difference; RR, relative ratio; SMD, standardized mean difference; WMD, weighted mean difference.

### 3.4 Primary outcome

#### 3.4.1 NIHSS

**FIGURE 4:**
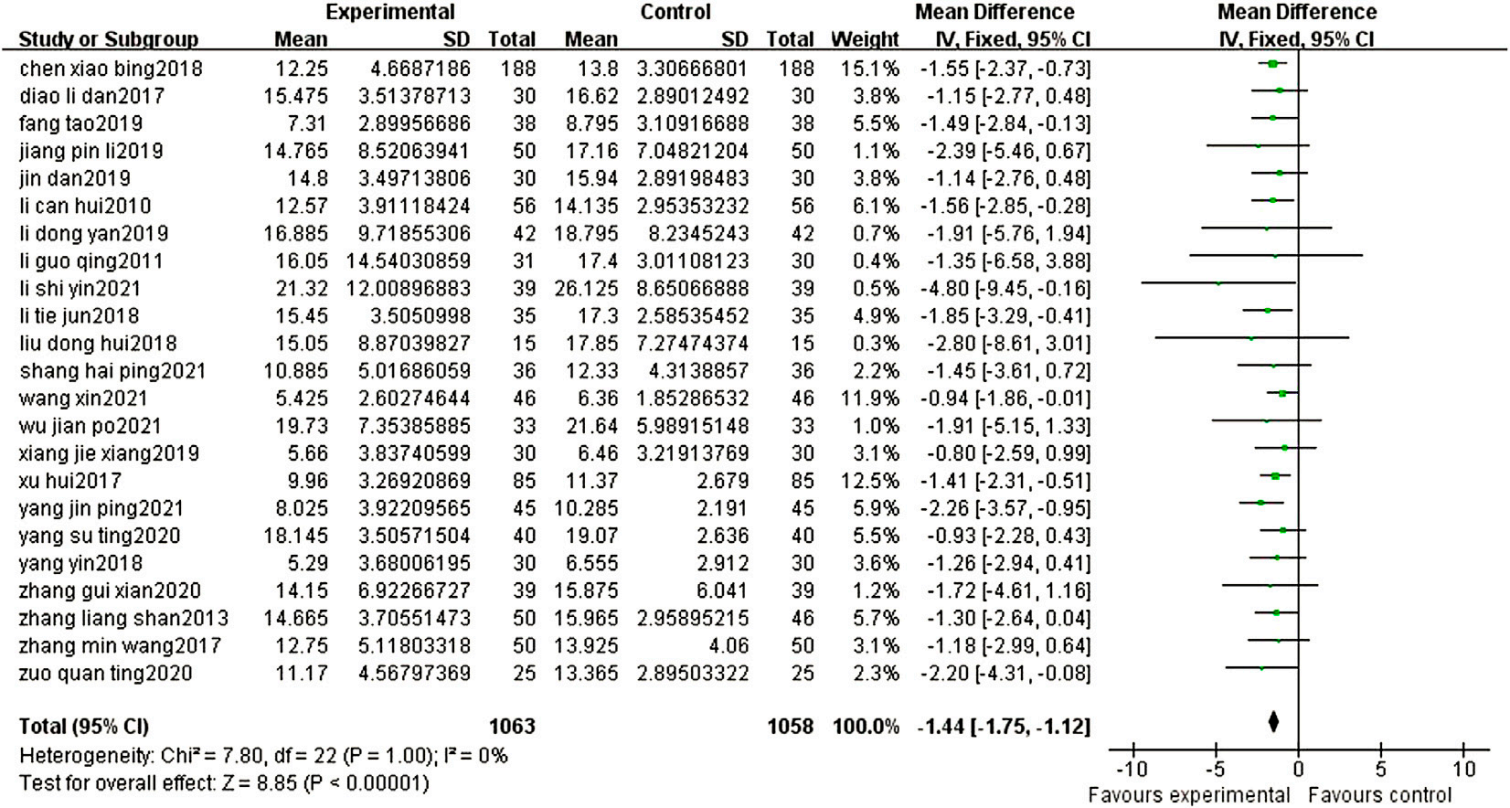
Forest plots showed that the NIHSS score of the experimental group decreased. Compared with that of the control group.

Twenty-three articles reported the NIHSS scores of patients after BYHWD combined with conventional treatment or conventional treatment alone. Due to the results of the heterogeneity test among the studies (*p* = 1.00, I^2^ = 0%), a fixed effect model was used. The results of the meta-analysis showed that the NIHSS score of the experimental group was significantly lower than that of the control group (MD = -1.44%, 95% CI: 1.75, -1.12, *p* < 0.00001) (Figure 4).

### 3.5 Secondary outcomes

#### 3.5.1 CSS

**FIGURE 5:**
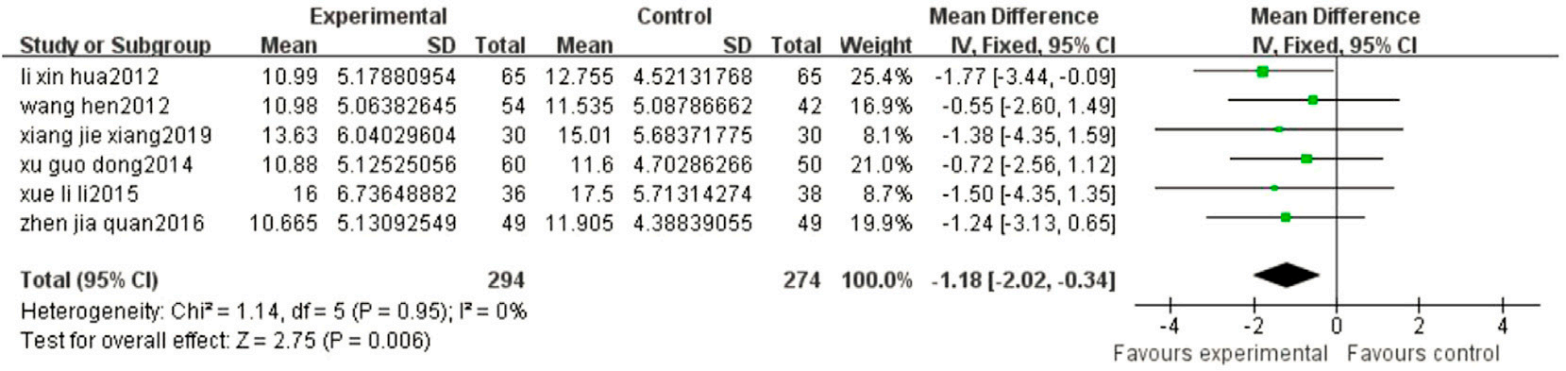
Forest plots showed that the CSS score of the experimental group decreased compared with that of the control group.

Six articles reported the CSS of patients after different treatments with BYHWD plus conventional treatment or conventional treatment alone. There was no heterogeneity among the studies (*p* = 0.95, I^2^ = 0%), and a fixed effect model was used. The results of the meta-analysis showed that the CSS score of the experimental group was statistically lower than that of the control group (MD = -1.18, 95% CI: 2.02, -0.34, *p* = 0.006) (Figure 5).

### 3.6 ADL

**FIGURE 6:**
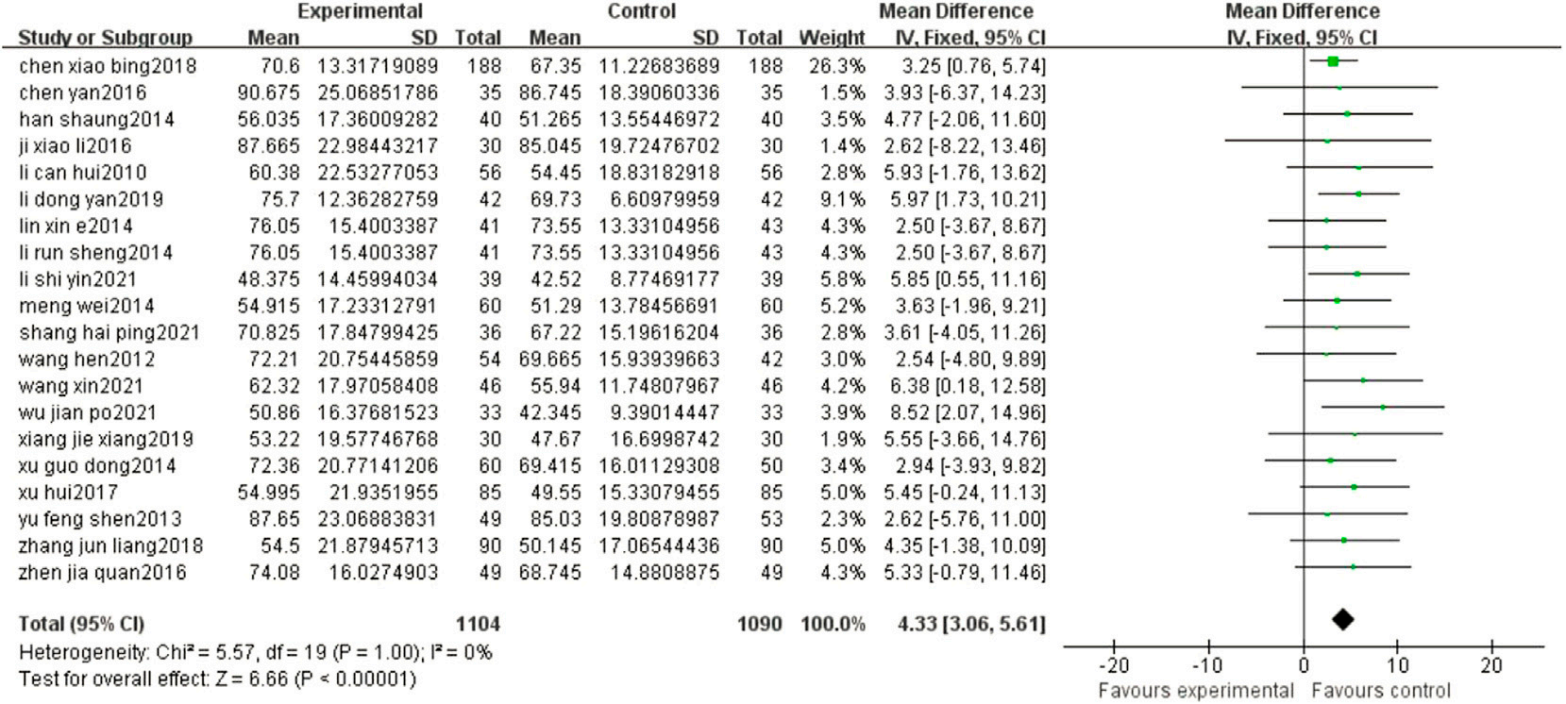
Forest plots showed that there was a significant improvement in the ADL in the experimental group compared with that of the control group.

The results of the meta-analysis showed that compared with the control group, the ADL of patients in the experimental group was significantly improved (MD = 4.33, 95% CI: 3.06, 5.61, *p* < 0.00001) (Figure 6).

### 3.8 ADR

**FIGURE 7:**
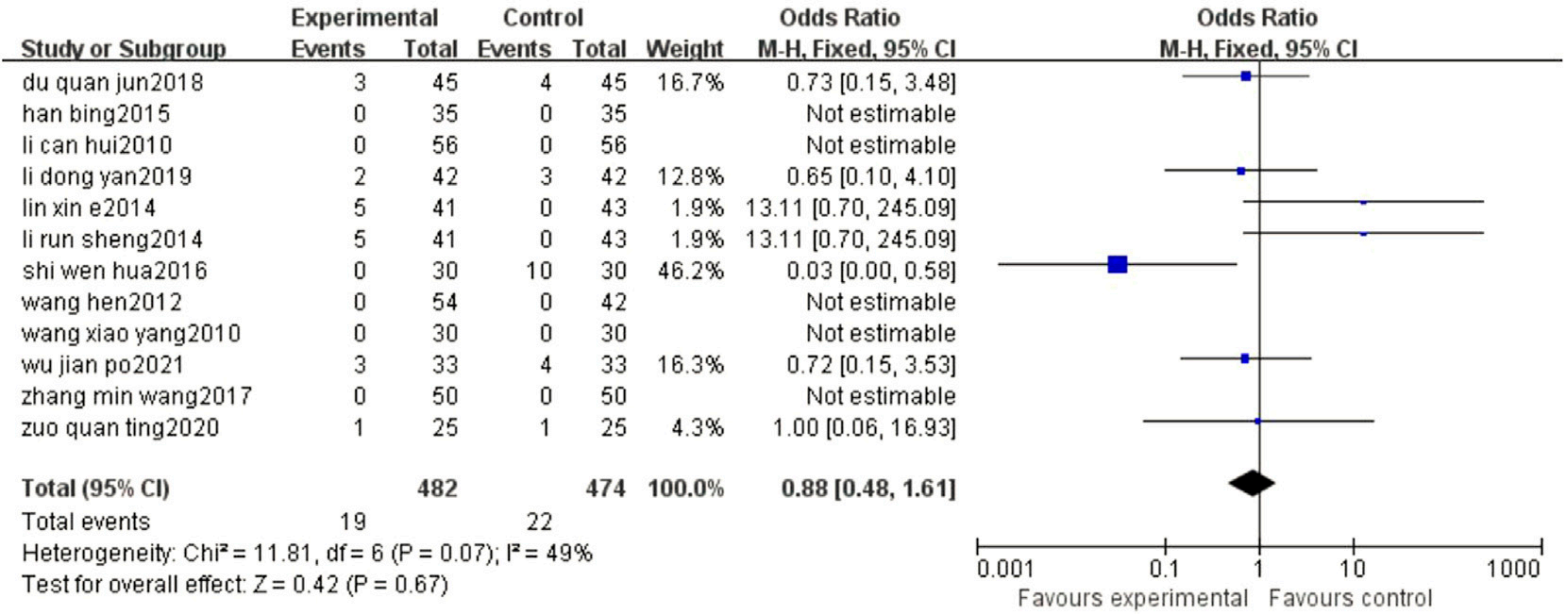
Forest plots showed that the increase in ADR in the experimental group was not obvious compared with that of the control group.

The results of the meta-analysis showed that compared with conventional treatment alone, BYHWD plus conventional treatment did not increase the adverse reactions of patients (OR = 0.88, 95% CI: 0.48, 1.61, *p* = 0.67) (Figure 7).

### 3.9 Publication bias

**FIGURE 8:**
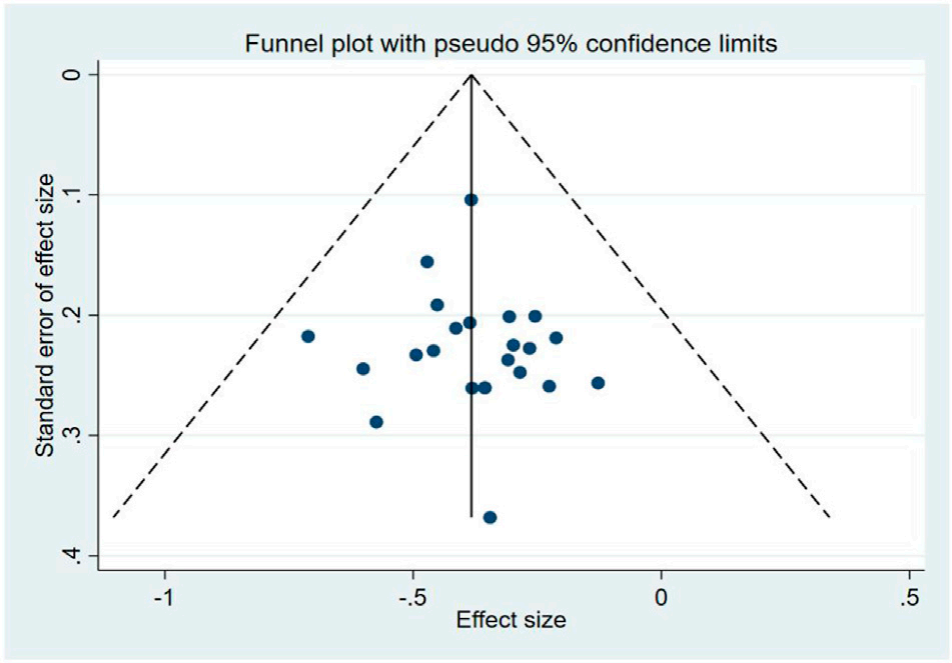
Funnel plots of NIHSS scores showed that there was no obvious publication bias.

The funnel plots of the NIHSS suggested that there was a possible publication bias in small trials (Figure 8). Egger’s test also indicated there was significant publication bias (*p* = 0.672).

### 3.10 Quality of evidence

Using GRADE, we assessed the certainty of the evidence to be moderate to low for outcomes for which data were available. In all 39 trials, the quality of evidence was downgraded by one level because of the unclear risk of method bias in some trials. The total number of patients was enough for each outcome, and the statistical heterogeneity of the results was small, so the quality of evidence for these outcomes was upgraded by one level. Consequently, the quality of evidence was moderate for the NIHSS, ADL, and ADR scores (Table 6).

**TABLE 6:** Evidence GRADE profile.

Outcome (no. Of trials)	Quality assessment						Sequelae of stroke				Clinical efficacy and safety	Quality
	Risk of bias	Inconsistency	Indirectness	Imprecision	Publication bias		BYHWD and CT	CT		95% CI	Adverse reactions	
NIHSS	None^b^	No	No	No	None^b^		1063/2121 (50.1%)	1058/2121 (49.9%)		-1.75 to -1.12	None	⊕⊕⊕Ο
Moderate												
CSS	None^b^	No	No	No	None^b^		294/568(51.8%)	274/568(48.2%)		-2.02 to -0.34	None	⊕⊕⊕Ο
Moderate												
ADL	None^b^	No	No	No	None^b^		1104/2194 (50.3%)	1090/2194 (49.7%)		3.06 to 5.61	None	⊕⊕⊕Ο
Moderate								474/956(49.6%)				
ADR	None^b^	No	No	No	None^b^		482/956(50.4%)			0.88 to 1.61	None	⊕⊕⊕Ο
Moderate												

**NIHSS:** national institute of health stroke scale; **CSS:** cincinnati stroke scale; ADL**:** activities of daily living; **ADR:** adverse drug reaction.

a Most trials had an unclear risk of methodological bias. Evidence was therefore downgraded by one level.

b Publication bias was not presented. The results were robust. Therefore, the evidence was not downgraded.

## 4 Discussion

Stroke is the main cause of disability and the second leading cause of death in the world (Paul and Candelario-Jalil, 2021). Ischemic stroke has become a global health problem that seriously threatens human life and health (Jiang et al., 2020). With the continuous development of medicine, the methods of treating ischemic stroke are also increasing, and TCM treatment has always played an important role in it. BYHWD is a classical Chinese medicine prescription for the treatment of ischemic stroke in the recovery period and has a good clinical effect on ischemic stroke in the recovery period (Liu et al., 2022). Therefore, this meta-analysis aimed to evaluate the clinical efficacy and safety of BYHWD in the recovery period of ischemic stroke patients.

A total of 39 studies involving 3,683 patients were included in this meta-analysis, and BYHWD combined with conventional treatment and conventional treatment alone were compared in patients with ischemic stroke in the recovery period. Under normal circumstances, clinical ischemic stroke patients often leave symptoms of different degrees of neurological deficits. Improving the symptoms of this neurological deficit and improving the activities of daily living have always been the top priorities in the treatment of cerebral infarction (Xing and Bai, 2020). Therefore, in this study, the NIHSS was the primary outcome measure, and CSS, ADR, and ADL were the secondary outcomes. The above indicators were used as clinical trial observation and efficacy evaluation indicators. The National Institute of Health Stroke Scale (NIHSS) score (Yamal 2021), which is a quantitative indicator of the severity of the stroke, is often used as a surrogate endpoint in clinical research and stratifies patients according to the NIHSS score to guide clinical decision-making. Effective treatment was defined as a decrease in the NIHSS score by more than four points or complete disappearance of symptoms after treatment. The CSS score includes horizontal gaze, level of consciousness, speech, limb flexibility, and walking ability. The higher the score, the worse the patient’s condition and the worse the neurological function (Cai and Zhang, 2022). The results of this study show that, compared with conventional treatment alone, BYHWD combined with conventional treatment can reduce the NIHSS score and CSS score of patients, suggesting that BYHWD can improve the neurological function of ischemic stroke patients in the recovery period. In terms of activities of daily living, the activities of daily living in the experimental group were stronger than those in the control group. Moreover, the results of the meta-analysis indicated that compared with the control group, the treatment of the experimental group in the recovery period of ischemic stroke did not increase the adverse reactions of patients. All the results prove that BYHWD is an effective therapy to improve the recovery period of ischemic stroke, which is beneficial for relieving the patient’s condition, promoting the improvement of the patient’s neurological function, and improving the quality of life without increasing adverse reactions.

BYHWD comes from Wang Qingren’s “Yilin Correction” in the Qing Dynasty. It is a commonly used prescription for the treatment of ischemic stroke. “This prescription treats hemiplegia, crooked eyes, slurred speech, salivation at the corners of the mouth, dry stools, frequent urination, and incontinence of enuresis” (Wu, 2019). This prescription has the compatibility characteristics of “not to remove blood stasis to activate blood, but to invigorate Qi to activate blood” (Liang et al., 2021). *Astragalus* trimestris L is the monarch drug in BYHWT, which has the effects of nourishing vitality, promoting blood circulation, and removing blood stasis; Angelica sinensis (Oliv.) Diels is the ministerial drug of the formula, which can activate blood and nourish blood and remove blood stasis; Other botanical drugs have activities of expectorating phlegm and dredging collaterals. The combination of the above drugs can have a synergistic effect and achieve the effects of nourishing Qi and promoting blood circulation, removing blood stasis, and dredging collaterals. As a popular traditional Chinese medicine formula, BYHWD was widely used for treating ischemic diseases. However, there are few studies focused on the effects of BYHWD on neurodegenerative diseases, and the underlying molecular mechanisms are largely elusive. Li Z *et al.* established a neurotoxic model in PC12 cells and adopted an innovative experimental grouping method to investigate the neuroprotective effects of BYHWD on neurotoxicity induced by 6-Hydroxydopamine (6-OHDA) exposure. They found that BYHWD had neuroprotective effects against the 6-OHDA-induced neurotoxicity *via* Akt/GSK3β pathway based on serum pharmacology methodology. (Li et al., 2016). Another study found that BYHWD could modulate multiple signaling pathways including the Jak/Stat3/cyclin D1 signaling pathway, EGFR/PI3K/Akt/Bad/14–three to three signaling pathway, caveolin-1, and Hes1. The modulations of these cellular signaling pathways contributed to the anti-apoptotic cell death, improvement of the neural stem cell proliferation, astrogenesis, and neurogenesis in post-ischemia brains, subsequently inducing the recovery of the neurological functions in the post-ischemic brains (Chen X. et al., 2020). In addition, modern studies have shown that BYHWD can reduce cerebral infarct size and improve neurological deficits in ischemic stroke rats and attenuate neuronal damage in rats with cerebral ischemia/reperfusion (I/R) injury (Li et al., 2021); BYHWD can promote neurogenesis and angiogenesis in rats with cerebral ischemia (Zhuge et al., 2020); BYHWD can protect the integrity of the neurovascular unit and improve the permeability of the blood-brain barrier, thereby improving stroke caused by cerebral ischemia (Zheng et al., 2021). Therefore, BYHWD can effectively treat ischemic stroke and can be widely used in the clinical treatment of ischemic stroke.

Limitations of this study: 1) Although the included trials were described as “random grouping”, most of the trials did not describe specific grouping methods, blinding, allocation concealment, *etc.*, so the possibility of selection bias cannot be ruled out; 2) Samples of most included studies were relatively small; 3) The efficacy evaluation of most studies was subject to a certain degree of subjectivity, and there was a lack of standard quantitative research; 4) Due to generally low quality of the included trials, this study can only draw very limited conclusions. There is an urgent need to improve the quality of the design and report of such studies.

## 5 Conclusion

Compared with conventional treatment alone, BYHWD combined with conventional treatment contributed to a significant improvement in clinical efficacy, neurological function, and activities of daily living, while it did not increase adverse reactions. Due to the limitations of this study, the quality of the included trials was generally low. In the future, more clinical trials with standardized designs, strict implementations, and large samples are needed to further verify the clinical efficacy and safety of BYHWD in the treatment of ischemic stroke in the recovery period and provide a more reliable evidence-based basis for clinical application.

## Data Availability

The original contributions presented in the study are included in the article/supplementary material, further inquiries can be directed to the corresponding authors.

## Abbreviations

CT, conventional treatment; NIHSS, National Institute of Health Stroke Scale; CSS, Chinese Stroke Scale; TCM, traditional Chinese medicine; ADL: activities of daily living; ADR, adverse drug reaction; RCT, randomized controlled trial; T/C, treatment group/control group.
